# Correction to *Lancet Child Adolesc Health* 2021; 5: 178–89

**DOI:** 10.1016/S2352-4642(25)00158-0

**Published:** 2025-05-16

*Cooper M, Stafford MR, Saxon D, et al. Humanistic counselling plus pastoral care as usual versus pastoral care as usual for the treatment of psychological distress in adolescents in UK state schools (ETHOS): a randomised controlled trial.* Lancet Child Adolesc Health *2021; **5:** 178–89*—In this Article, the publication date should have been January 20, 2021. This correction has been made to the online version as of May 16, 2025.

